# Antimicrobial effect of Red Roselle (Hibiscus Sabdariffa) against different types of oral bacteria

**DOI:** 10.25122/jml-2021-0184

**Published:** 2022-01

**Authors:** Abeer Abdulridha Abass, Mohanad Jameel Najm Al-Magsoosi, Wijdan Abdulameer Kadhim, Ruba Mustafa, Sana’a Abdulrazzaq Ibrahim, Abtesam Imhemed Aljdaimi, Suhad Jabbar Al-Nasrawi, Najah Raiesh Hadi, Julfikar Haider

**Affiliations:** 1. Department of Basic Science, Faculty of Dentistry, Kufa University, Najaf, Iraq; 2. Department of Oral Diagnosis, College of Dentistry, University of Basrah, Basrah, Iraq; 3. Department of Conservative Dentistry, College of Dentistry, University of Karbala, Karbala, Iraq; 4. Department of Conservative Dentistry, Faculty of Dentistry, Jordan University of Science and Technology, Irbid, Jordan; 5. College of Dentistry and Oral Surgery, Alasmarya University, Zliten, Libya; 6. Department of Conservative Dentistry, Faculty of Dentistry, Kufa University, Najaf, Iraq; 7. Department of Pharmacology & Therapeutics, Faculty of Medicine, University of Kufa, Najaf, Iraq; 8. Department of Engineering, Manchester Metropolitan University, Manchester, United Kingdom

**Keywords:** Hibiscus Sabdariffa, Red Roselle, *Streptococcus mutans*, *Staphylococcus aureus*, *Enterococcus faecalis*, intra canal irrigant, mouthwash, RE – an aqueous extract Red Roselle calyx, CH – Chlorhexidine, ACA – Amoxicillin-clavulanic acid, Tet – Tetracycline, Met – Metronidazole, *S. mutans* – *Streptococcus* *mutans*, *S. aureus* – *Staphylococcus aureus*, *E. faecalis* – *Enterococcus* *faecalis*, BIZ – Bacterial inhibition zones, MIC – Bacterial minimum inhibitory concentration, BMDM – Broth Micro dilution method

## Abstract

This study aimed to compare the antimicrobial effect of an aqueous extract Red Roselle calyx (RE), Chlorhexidine (CH), Amoxicillin-clavulanic acid (ACA), Tetracycline (Tet), and Metronidazole (Met)on *Streptococcus mutans* (S. mutans), *Staphylococcus* *aureus* (*S. aureus*) and *Enterococcus faecalis* (*E. faecalis*) bacteria. The bacterial inhibition zones (BIZ)of the RE (25, 50, 75, 100) mg/ml and CH solutions (0.2%, 2%) were determined using the agar well diffusion method. Additionally, the susceptibility of the tested bacteria against (30 μg) of standard antibiotics of ACA, Tet, and Met was examined. The bacterial minimum inhibitory concentration (MIC) was measured using the Broth Micro dilution method (BMDM). All tests were carried out in triplicates, and water was considered the negative control. For *S. mutans*, the RE at 50 mg/ml or above concentrations displayed higher BIZ than 0.2% CH. 100 mg/ml of RE recorded a greater BIZ than the 2% CH. The greater BIZ against S. mutans was recorded by Tet. A comparable effect was found with 0.2% CH (75, 100) mg/ml of the RE against *S. aureus*. Greater BIZ for *S. aureus* and *E. faecalis* were reported for 100 mg/ml RE compared to the Tet and Met RE at 100 mg/ml inhibited the *E. faecalis* growth in a zone size comparable to the CH (0.2%, 2%).The RE with 50,100 mg/ml concentrations showed comparable antimicrobial effect to 0.2%, 2% concentrations of CH, respectively. As an herbal substitute for commercial disinfectants, the RE can be considered an effective final endodontic irrigant and dental mouthwash.

## Introduction

Microorganisms are a major etiological factor for many diseases, where a specific microorganism may harbor a site in the human body, then grow, colonize, and release its toxins [1]. Oral pathogens play the most important role in inducing dental caries, periodontal diseases [2], and endodontic treatment failure and retreatment [3]. Pathogenic species of *S. mutans*, broadly classified as the main causative agent of dental decay, are gram-positive bacteria. However, other bacteria like Actinomycesand lactobacillus might be involved in causing the dental caries process. These strains accumulate and adhere to the surface of the tooth producing extracellular polysaccharides, which are essential for biofilm formation [4]. *S. aureus* is one of the main causative agents of human infection, with severity ranging from mild to severe fatal. It plays a potential role in a number of oral diseases [5]. *E. faecalis* is one of the main pathogens responsible for endodontic failure during endodontic treatment since it is highly resistant to disinfectant agents. For instance, *E. faecalis* can survive in different environments in inaccessible areas for chemo-mechanical debridement, create a biofilm, have resistance to various disinfectants, and synergistic reactions with different bacterial strains. Thus, alternative disinfectants have to be explored to overcome the activities of microbial pathogens in the root canal system [3]. Chlorhexidine (CH) solution has been recommended as a mouthwash (0.2%), an endodontic irrigant, or root canal medicament (2%). It has a broad-spectrum antibacterial activity with prolonged gradual release at therapeutic levels. However, recently, it has been reported that the prolonged use of CH mouthwash has side effects such as staining, development of hypersensitivity reactions, causing major changes in the salivary microbiome after 7 days of use which increases caries development [6]. It could cause an inflammatory reaction to soft oral tissue [7], which restricts the application of CH [8]. For infectious diseases, several antibiotics nowadays are available. However, they have limited use due to drug toxicity, low potency, poor solubility, and the development of resistant strains [9–10].

Thus, a great effort to develop new antibacterial agents is a fundamental need to replace synthetic antibiotics. The utilization of herbal products is considered the best alternative to synthetic antibiotics due to their natural and non-narcotic properties with affordable price, no adverse side effects, and no impact on the environment [11]. In fact, ancient herbal medicines are considered the source of many modern medicines. Therefore, plant extracts can be a promising source of novel antimicrobial agents [12]. Red Roselle is a medical plant with worldwide fame, and its scientific name is Hibiscus Sabdariffa. It has many medically important contents, named phytochemical, with well-known medical and nutritional values [13]. The reported phytoconstituents are responsible for bacterial inhibition. The antibacterial effectiveness of RE against pathogenic bacteria such as Bacillus cereus, *Staphylococcus aureus* [14–15], Shigella flexneri, Salmonella, Listeria monocytogenes, Typhimurium [16–18], Escherichia coli [19–20], and Bacillus anthracis [21] was demonstrated in the literature. Only a few researchers have tested the impact of RE as an antibacterial agent in the dental field, such as a mouthwash against *S. mutans* and *S. aureus* [8]. In addition, the ability of RE to inhibit the growth of *E. faecalis as* an endodontic intracanal irrigant is still uncertain. As a result, this is the first study to examine the antimicrobial effect of the RE against *E. faecalis*. Furthermore, its antimicrobial activity was compared to CH, ACA, Tet, and Met as these antibiotics are widely used in the dental field [22, 23]. The null hypothesis was that there would be a significant difference in BIZ caused by the tested RE at different concentrations, CH, and the selected antibiotics (ACA, Tet, and Met).

## Material and Methods

**Figure 1.**
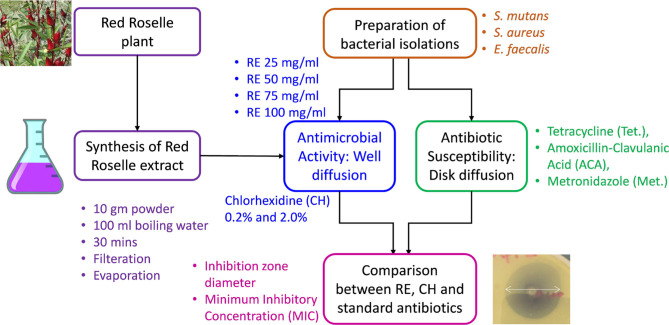
Methodology adopted for evaluating the antibacterial effect of Red Roselle Extract (RE).

Figure 1 presents the general workflow followed in the research work to evaluate the effectiveness of RE as an antimicrobial agent.

### Plant samples and preparation of extracts

The fresh red Roselle plant was obtained from the botanic garden in Najaf, Iraq; the red calyxes of Hibiscus Sabdariffa were separated from the plant and dried for a week at 25°C. The dried calyxes were ground to powder, kept in a sealed container, and stored in a refrigerator (4°C) until used. Aqueous Roselle calyx extract was prepared by adding 10 gm of the previously prepared powder to 100 ml of boiling water and then heated on a hot stirrer plate for 30 min. To remove the remnants, the mixture was filtered via Whatman No 1 filter paper (Whatman products, Springfield Paper Mill, Maidstone, UK). The water content of the filtered solution was evaporated using an air recirculation oven and then kept at 4°C in the dark until used to determine antibacterial effectiveness [8].

### Bacterial strains

Cultures of *S. mutans*, *S. aureus*, and *E. faecalis* were prepared utilizing blood agar and mannitol salt agar (Difco, USA) and incubated at 37°C for 18–24 hrs. For bacterial identification, the same techniques were used according to the Bergeys Manual and VITEK2 System [24]. The tested bacterial samples were donated by the Microbiology Lab of the Faculty of Dentistry at the University of Kufa.

### Determination of Antibiotic Susceptibility

Muller-Hinton agar medium (Oxoid, UK) was employed to examine the susceptibility of *S. mutans*, *S. aureus*, and *E. faecalis* via the disk diffusion method. The disks of ACA (30 μg), Tet (30 μg), and Met (30 μg) (Himedia, India) were applied as the standard antibiotic. MacFarland tube number 0.5 was involved as standard for inoculums preparation which approximately contained 1×10^8^ CFU/ml. After overnight incubation at 37oC, according to Baur *et al.* [25], the areas of inhibition were measured and interpreted as the National Committee for Clinical Laboratories Standard guidelines [26].

### Antimicrobial activity of RE calyx extract and CH (0.2% and 2%)

#### Well diffusion method

Antibacterial activities were determined using an agar well diffusion method [27]. The isolated pathogens were suspended in sterile water and diluted to 10^8^ CFU/ml. The inoculums were spread by sterile cotton swabs onto the surface of the Mueller Hinton agar. After 15 min, wells (8 mm in diameter) were cut from the agar using a sterilized cork borer. Using a micropipette, an equal volume (100 μl) of 100, 75, 50, and 25 mg/ml of Roselle Calyces’ aqueous extract and CH (0.2% and 2%) was poured separately onto the wells. The 0.2% CH indicated 0.002 mg/ml, and the 2% CH indicated 0.02 mg/ml. The CH plates were considered the positive control, while sterile water was used as the negative control. Both the positive and negative controls were left at 37°C for overnight incubation. The antibacterial activity was evaluated by measuring the inhibition zones. All tests were conducted in triplicate.

#### Determining minimum inhibitory concentration

The Broth Micro dilution method (BMDM) was used to assess bacterial minimum inhibitory concentration (MIC) [28]. Briefly, 100 μl of serial doubling dilutions of the aqueous extract was added with a pipette in a 96-well microtiter plate ranging from 0.28 to 128 mg/ml, and 100 μl of Muller-Hinton broth were added to each well. They were prepared in triplicate. Finally, 100 μl of bacterial suspension (10^6^ CFU/ml) was mixed in each well to achieve 10^4^ CFU/ml in each well. The plates were covered to prevent dehydration, and they were incubated at 37°C for 18–24 hours. The turbidity of growth was evaluated visually. The lowest concentration at which the turbidity changed was considered the MIC value, which inhibited bacterial growth [29]. Negative control was sterile water, and positive control was 2% CH.

### Statistical analysis

Statistical analysis was performed using SPSS software version 22.0 (IBM Corp., Armonk, USA). Results were expressed as mean and standard deviation. As the data were not normally distributed, the significance of the differences between the groups was determined using the Mann-Whitney U test, where the 0.2% CH then 2% CH was considered as the control group in the comparison of the BIZ, then the 100 mg/ml RE was compared to the antibiotics. The 2% CH was again considered the control group in comparing the MIC. The data of MIC was normally distributed, so it was compared using the independent samples t-test. All the tests were conducted at a confidence level of 95% and P<0.05.

## Results

**Figure 2.**
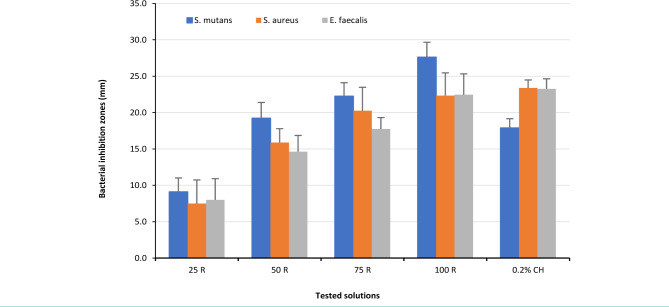
Mean values of Bacteria Inhibition Zone (BIZ) diameters caused by RE at different concentrations 25 (25 R), 50 (50 R), 75 (75 R) and 100 (100 R) mg/ml) with 0.2% CH.

**Figure 3.**
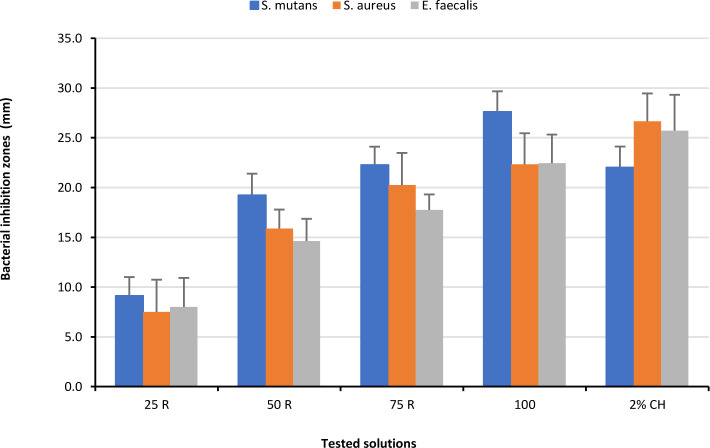
Mean values of Bacteria Inhibition Zone (BIZ) diameters caused by RE at different concentration 25 (25 R), 50 (50 R), 75 (75 R) and 100 (100 R) mg/ml) with 2% CH.

**Figure 4.**
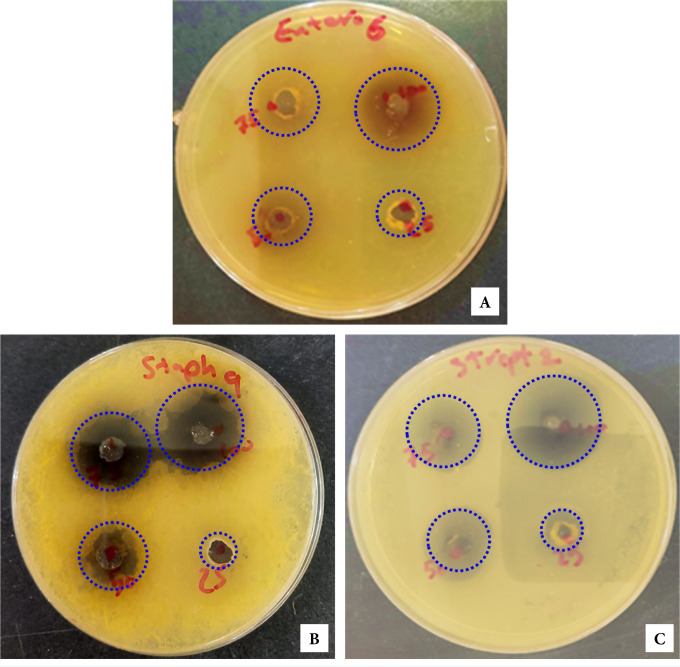
Inhibition zone of bacterial isolates of various concentrations (25, 50, 75, and 100 mg/ml) of RE against (A) S. mutans, (B) S. aureus (where a dual action of the extract is clear), and (C) E. faecalis.

The antimicrobial activity results of four different RE concentrations (25, 50, 75 and 100 mg/ml) with 0.2% and 2% CH were evaluated against three selected bacteria isolates, table 1, table 2, and figures 2–4. All the samples of the negative control recorded no inhibition (0 mm). Compared to the result of the negative control, the RE for all tested concentrations showed marked inhibition zones against all three tested bacteria. The 25 mg/ml RE recorded the smallest BIZ, while 100 mg/ml RE showed the largest zone. In comparison to the effect of 0.2% CH against *S. mutans*, the 50 mg/ml, 75 mg/ml and 100 mg/ml RE showed larger bacterial inhibition zones (BIZ) but significant differences were recorded between the 75 mg/ml and 100 mg/ml concentrations and the 0.2% CH. Concerning *S. aureus*, the 0.2% CH recorded the largest inhibition zones, closely followed by 75 mg/ml and 100 mg/ml with no statistically significant differences. Again, the 0.2% CH showed the largest inhibition zones against the *E. faecalis*, closely followed by 100 μg/ml with no statistically significant difference. Other RE concentrations showed significantly lower BIZ.

**Table 1.** Mean and standard deviation of the BIZ (mm) of RE at different concentrations compared to 0.2% CH.

**Bacterial Isolates**	**Mean of BIZ (SD) at different concentrations of RE**				**Mean BIZ diameters (mm) CH (0.2%)**
	**25 mg/ml**	**50 mg/ml**	**75 mg/ml**	**100 mg/ml**	
**S. mutans**	9.1 (1.9) * (p=0.000)	19.2 (2.1) (p=0.094)	22.2 (1.9) * (p=0.000)	27.6 (2.1) * (p=0.000)	17.9 (1.3)
**S. aureus**	7.5 (3.2) * (p=0.000)	15.9 (1.9) * (p=0.000)	20.2 (3.2) (p=0.094)	22.3 (3.1) (p=0.094)	23.4 (1.1)
**E. faecalis**	8.0 (2.9) * (p=0.000)	14.6 (2.2) * (p=0.000)	17.8 (1.6) * (p=0.000)	22.4 (2.9) (p=0.340)	23.3 (1.4)

* – indicated a statistically significant difference in the Mann-Whitney U test.

**Table 2.** Mean and standard deviation of the BIZ (mm) of RE at different concentrations compared to 2% CH.

**Bacterial Isolates**	**Mean of BIZ (SD) at different concentrations of RE**				**Mean BIZ diameters (mm) CH (2%)**
	**25 mg/ml**	**50 mg/ml**	**75 mg/ml**	**100 mg/ml**	
**S. mutans**	9.1 (1.9) * (p=0.00)	19.2 (2.1) * (p=0.008)	22.2 (1.9) (p=0.605)	27.6 (2.1) * (p=0.00)	22.0 (2.1)
**S. aureus**	7.5 (3.2) * (p=0.00)	15.9 (1.9) * (p=0.00)	20.2 (3.2) * (p=0.001	22.3 (3.1) * (p=0.011)	26.7 (2.8)
**E. faecalis**	8.0 (2.9) * (p=0.00)	14.6 (2.2) * (p=0.00)	17.8 (1.6) * (p=0.00)	22.4 (2.9) (p=0.050)	25.7 (3.6)

* – indicated a statistically significant difference in the Mann-Whitney U test.

Regarding the effect of 2% CH against *S. mutans*, the 100 mg/ml RE showed significantly larger BIZ with non-significant differences between the 75 mg/ml RE and the 2% CH. In relation to *S. aureus*, the 2% CH recorded the largest BIZ, followed by 100 μg/ml, then 75 mg/ml RE without any significant difference. The 2% CH revealed the largest BIZ against the *E. faecalis*, closely followed by 100 mg/ml RE with no statistically significant difference, then the rest of the extracts with the concentrations in a descending order showed significantly lower BIZ values. In relation to the RE concentration, as shown in Figure 4, the greater the concentration the greater the inhibition zone against *S. mutans* (Figure 4a), *S. aureus* (where a dual action of the extract is clear, Figure 4b), and *E. faecalis* (Figure 4c).

**Figure 5.**
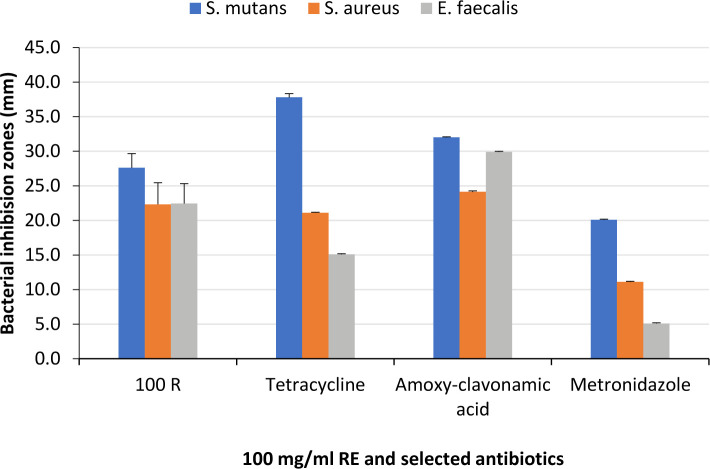
Mean values of BIZ diameters with 100 mg/ml RE compared to the selected antibiotics.

Table 3 and Figure 5 show a comparison of BIZ diameters measured from the tests using 100 mg/ml of RE, Tet, ACA, and Met. The results showed the largest BIZ diameter gains *S. mutans* with Tet. The ACA also recorded a significantly larger zone than 100 mg/ml RE, while the Met recorded significantly smaller BIZ. On the other hand, ACA generated the biggest BIZ diameter against *S. aureus* and *E. faecalis*, followed by 100 mg/ml RE, then the Tet, while the Met recorded the smallest diameters.

**Table 3.** The mean and standard deviation of the BIZ (mm) of 100 mg/ml RE and the selected antibiotics.

**Bacterial Isolates**	**Mean and standard deviation of BZI (SD)**			
	**100 mg/ml of RE**	**30 μgTet.**	**30 μgACA**	**30 μgMet.**
**S. mutans**	27.6 (2.1)	37.8 (0.5) * (p=0.000)	32.0 (0.1) * (p=0.000)	20.1 (0.1) * (p=0.000)
**S. aureus**	22.3 (3.1)	21.1 (0.1) (p=0.605)	24.1 (0.1) (p=0.05)	11.1 (0.1) * (p=0.000)
**E. faecalis**	22.4 (2.9)	15.1 (0.1) * (p=0.000)	29.9 (0.1) * (p=0.000)	5.1 (0.1) * (p=0.000)

* – indicated a statistically significant difference in the Mann-Whitney U test.

As shown in table 4, generally, the tested bacteria required a lower concentration of CH than RE to be inactivated. In comparison to the other bacteria, a lower concentration of the extract was required to inhibit the growth of *S. mutans* and *E. faecalis*. Concerning the MIC of CH, the *S. aureus* required a comparatively higher concentration.

**Table 4.** Minimum inhibitory concentrations of RE and CH.

**Bacteria**	**MIC for CH (mg/ml)**	**MIC for RE (mg/ml)**
**S. mutans**	0.008 (0.0) *	32 (0.0)
**S. aureus**	0.016(0.0) *	32 (0.0)
**E. faecalis**	0.008 (0.0) *	16 (0.0)

* – indicated a statistically significant difference in the independent samples t-test.

## Discussions

In the current study, the possible antibacterial activity of RE against the *S. mutans*, *S. aureus*, and *E. faecalis*, was investigated and compared with respect to standard antiseptic (CH) and antibiotics(ACA, Tet, and Met). The RE showed a marked antibacterial activity against all the selected types of bacteria with the highest activity at 100 mg/ml, recording the greatest reduction against the *S. mutans*, then *E. faecalis*, and *S. aureus*. Therefore, the tested hypothesis was rejected.The effect of the 100 mg/ml concentration of RE on the *E. faecalis* in the present study was comparable to that of 2% CH. To the best of our knowledge, there was no available data in BIZ for RE on *E. faecalis* to compare with this current finding. In addition, no study analyzed the MIC of the tested extract against the *S. mutans* or *E. faecalis*. In relation to the MIC of the CH, the current study recorded 0.008, 0.016, and 0.008 (mg/ml) against *S. Mutans*, *S. aureus*, and *E. faecalis*, respectively. The recorded value in previous study were 0.01024 mg/ml, 0.00064 mg/ml, and 0.00016 mg/ml against *S. mutans*, *S. aureus*, and *E. faecalis*, respectively [30], and 4–5 mg/ml in another study applying broth dilution method using tubes. Bermardo *et al.* [31] also reported higher MIC against *S. aureus* (0.122 mg/ml) using the well microplates method. In the literature, the MIC of the tested extract against *S. aureus* was between 25 and 50 mg/ml [32], and 20 and 10 mg/ml against both of *S. mutans* and *S. aureus*. While in this study, MIC was 32 mg/ml against both *S. mutans* and *S. aureus*. These differences could be attributed to the methodology used, such as performing the test by applying broth dilution method using tubes, while in the current study, the MIC test was performed using the well microplates method. In relation to the MIC of the RE against the *E. faecalis* this is the first study determining it is as low as 16 mg/ml. However, it is lower than required against the *S. mutans* or S. aureous. The antibacterial effectiveness of RE against *S. aureus* was in accordance with the previous studies [15, 33–36].

According to Sulaiman *et al.* [36], the RE inhibited the growth of *S. aureus* in inhibition zones ranging from 13.5, 13, 12, 8 mm by 200, 100, 50, 25 mg/ml, respectively.A marked inhibition zone (23 mm) was reported by RE at 100% mg/ml concentration against *S. aureus* [15], which was closer to that recorded by the present study (22.1 mm). This result was in agreement with the findings of previous studies. In the current study, although CH at 0.2% and 2% showed the largest BIZ against *S. aureus* and *E. faecalis*, the tested extract at 75, 100 mg/ml caused greater inhibition against *S. mutans*. That was supported by Baena-Santillán *et al.* [8] in their study when they compared the effect of 0.12% CH and RE against *S. mutans* and *S. aureus*. However, they recorded smaller BIZ (CH: 11 and 12.2 mm and RE: 10 and 9.5 mm against *S. mutans* and *S. aureus*, respectively). This could be attributed to the differences in the antibacterial testing technique as they used paper disc saturated with the extract, while an agar-well diffusion method was conducted in the present study. The antibacterial activity for RE can be attributed to the presence of phytochemical compounds [37] such as tannin, phenols, glycosides, terpenoids and saponins. These bioactive compounds exert their antimicrobial action by different mechanisms. Tannins are polymeric phenolics divided into two main categories: hydrolyzable and condensed tannins [38]. Its biological activity is interrelated to oxidation and polymerization patterns [39]. The antimicrobial effect is exerted by interacting with proteins through covalent and non-covalent interactions. In addition, they could make a compound with polysaccharides. Tannins have the ability to bind the cell walls of bacteria, inhibiting growth and protease activity. It also interacts with the microbial proteins, thus making the nutritional proteins unavailable for microbial growth [40]. Different mechanisms are supposed to be accountable for the antimicrobial activity of the phenols, including enzyme inhibition via oxidation.

Moreover, some phenolics, such as quinones, are a source of stable free radicals which irreversibly bind with bacterial proteins resulting in their loss of function. Further mechanisms of antimicrobial activity could be expressed by inactivating enzymes, binding to cell wall proteins, and interacting with substrates, rendering them unavailable to the microorganism, complexing with metal ions, and others [38]. Furthermore, anthocyanins pigment, which is available in the calyxes of the Roselle, is rich in polyphenolic compounds and phenolic acids. According to the current study, the Tet and ACA demonstrated a more significant antibacterial effect than Met against the tested bacteria. Although ACA had the most effective antibacterial effect against *S. aureus* and *E. faecalis*, the Re at 100 mg/ml demonstrated a greater effect than Tet and Met against both the bacteria. It was reported that using an aqueous extract of 100 mg/ml RE against *S. aureus* was more effective than gentamicin [15, 32], Tet, ampicillin, penicillin, and cephalosporin [15]. However, no study compared the effect of RE with the ACA and Met against *S. aureus*, and the Tet, ACA, and Met against *S. mutans* and *E. faecalis*. One limitation of this study was that the effectiveness of the RE was tested on limited types of microorganisms. Therefore, further research is needed to assess the antifungal effect of the RE, as well as the role in inhibiting dental plaque and biofilm formation. A future clinical study was recommended to evaluate the effectiveness of RE in endodontic treatment under intraoral conditions. There could be a risk of tooth discoloration after using RE because of its red pigment, so it is advisable to have a final normal saline flash to overcome this issue.

## Conclusion

With the limitation of this in vitro study, the aqueous extract of RE with 50 mg/ml, 100 mg/ml concentrations showed comparable antimicrobial effects to 0.2%, 2% concentrations of CH, respectively. The RE at 75 and 100 mg/ml resulted in greater antibacterial effectiveness than Met against *S. mutans*, *S. aureus*, and *E. faecalis*. They were better than the Tet against the *E. faecalis*. The MIC for inactivation of *E. faecalis* could be as low as 16 mg/ml. The RE can be an effective alternative herbal substitute for final endodontic irrigant in retreatment or apical surgery and as dental mouthwash compared to the standard antiseptic or antibiotic.

Although CH is still one of the most common commercial oral rinse and root canal irrigant products, aqueous extract of RE may work as a natural and reliable substitute for commercial antimicrobial mouth rinse and endodontic irrigant to avoid the side effects of CH.

## Acknowledgments

### Conflict of interest

The authors declare no conflict of interest.

### Authorship

AAA and SJA contributed to conceptualizing the study. WAK, AAA, and SJA contributed to the methodology of the study. AAA, MJNA, AIA, and JH contributed to writing the original draft, NRH and JH contributed to editing the manuscript. RM contributed to data collection, SAI contributed to data curation, and SJA contributed to data analysis.
